# Visit-to-Visit Systolic Blood Pressure Variability and Risk of Ischemic and Hemorrhagic Stroke

**DOI:** 10.3390/medicina61020267

**Published:** 2025-02-04

**Authors:** Oana Elena Sandu, Carina Bogdan, Adrian Apostol, Larissa Madalina-Alexandra Daniluc, Amanda Claudia Schuldesz, Mihaela Adriana Simu

**Affiliations:** 1 Department VII, Internal Medicine II, Discipline of Cardiology, “Victor Babeş” University of Medicine and Pharmacy, Eftimie Murgu Sq. No. 2, 300041 Timisoara, Romania; oana.ciolpan@umft.ro (O.E.S.); adrian.apostol@umft.ro (A.A.); larissa.daniluc@umft.ro (L.M.-A.D.); 2 Cardiology Clinic, “Pius Brinzeu” Clinical Emergency County Hospital Timisoara, Bd. Iosif Bulbuca No. 10, 300723 Timisoara, Romania; 3 Department of Neurology, Victor Babes University of Medicine and Pharmacy Timisoara, Eftimie Murgu Sq. No. 2, 300041 Timisoara, Romania; amanda.schuldesz@umft.ro (A.C.S.); simu.mihaela@umft.ro (M.A.S.); 4 Department of Neurology, “Pius Brinzeu” Clinical Emergency County Hospital Timisoara, Bd. Iosif Bulbuca No. 10, 300723 Timisoara, Romania

**Keywords:** visit-to-visit systolic blood pressure variability, ischemic stroke, hemorrhagic stroke

## Abstract

*Background and Objectives*: Systolic blood pressure (SBP) variability has been increasingly associated with cardiovascular outcomes, including stroke. This study aimed to evaluate the association between visit-to-visit SBP variability and the risk of ischemic and hemorrhagic stroke. *Materials and Methods*: A prospective cohort study was conducted on a set of 208 hypertensive patients over a period of three years, from August 2021 to September 2024, at the County Emergency Hospital “Pius Brinzeu”, Timișoara. Patients included in the study were stroke-free. SBP variability was quantified as the standard deviation of SBP measurements obtained quarterly. *Results*: This study demonstrated that systolic blood pressure (SBP) variability serves as a robust predictor of stroke incidence, underscoring its important role in cerebrovascular risk. The study cohort had an average age of 65.3 ± 9.1 years, with 53.4% males and 46.6% females. Patients in the highest SBP variability group had a 1.21-fold increased risk (21%, *p* = 0.031) of ischemic stroke and a 1.73-fold increased risk (73%, *p* = 0.005) of hemorrhagic stroke compared to those in the lowest variability group, revealing that higher SBP variability is strongly associated with an increased risk of both ischemic and hemorrhagic strokes, with the relationship being particularly pronounced for hemorrhagic stroke. Patients exhibiting greater fluctuations in SBP experienced significantly earlier stroke events and reduced stroke-free survival. Moreover, mortality rates were notably higher among individuals with very high SBP variability, indicating its profound impact on long-term outcomes. *Conclusions*: Visit-to-visit SBP variability is a significant and independent predictor of both ischemic and hemorrhagic stroke, emphasizing the clinical importance of monitoring and managing blood pressure stability. Further research should explore interventions to mitigate SBP variability and its impact on cerebrovascular outcomes.

## 1. Introduction

High blood pressure (BP) is a major risk factor in stroke incidence and cardiovascular events [1,2], particularly for lacunar strokes and atherothrombotic infarctions [3], remaining a significant public health concern [4]. According to ESC guidelines, high blood pressure is defined as office systolic BP ≥ 140 mmHg or office diastolic BP ≥ 90 mmHg [5].

Elevated systolic blood pressure (SBP), in particular, exerts profound effects on the vascular system by promoting endothelial dysfunction and arterial stiffness, thereby increasing susceptibility to cerebrovascular accidents. SBP variability increases mechanical stress on the vascular system and accelerates pathophysiological mechanisms such as atherosclerosis [6]. Arboix et al. reinforce that the pathophysiology of hypertension disproportionately contributes to these subtypes of ischemic stroke due to its impact on arterial stiffness, endothelial dysfunction, and the development of small-vessel disease and atherosclerosis [3].

Although mean SBP has historically been the primary focus of blood pressure management, growing evidence underscores the prognostic importance of visit-to-visit SBP variability, which can be an even stronger predictor of adverse outcomes than average SBP alone [7,8,9,10]. 

High blood pressure variability has been shown to be a better predictor of all-cause and cardiovascular mortality, stroke, and cardiac disease compared to average systolic blood pressure [11,12]. Visit-to-visit SBP variability is particularly significant because it reflects not just the absolute blood pressure level but also the stability of blood pressure over an extended period [7,8,9,10,11,12,13].

The World Health Organization (WHO) defines stroke as “rapidly developing clinical signs of focal (or global) disturbance of cerebral function, with symptoms lasting 24 h or longer or leading to death, with no apparent cause other than vascular origin” [14].

Visit-to-visit SBP variability is associated with an increased risk of stroke, especially in patients where SBP variability was significantly increased compared with patients with a small baseline SBP variability [15]. Visit-to-visit SBP variability is an independent predictor of primary stroke in hypertensive patients, as shown by Men et al. [16].

In our prospective cohort study, we targeted patients diagnosed with high blood pressure who were known to be without prior strokes or atrial fibrillation history in order to investigate the association between SBP variability, stroke risk, and mortality while also accounting for other cardiovascular risk factors and comorbidities.

## 2. Materials and Methods

### 2.1. Population

Our study, conducted prospectively, included 247 patients previously diagnosed with high blood pressure. The study took place over a period of three years, from August 2021 to September 2024. The patients were evaluated at a three-month interval in the outpatient clinic of the Cardiology Department at County Emergency Hospital “Pius Brinzeu” in Timisoara.

Participants were excluded from the study if they had a prior diagnosis of atrial fibrillation (*n* = 23), strokes (*n* = 11), or both conditions (*n* = 5). 

Every participant provided documented informed consent in accordance with the ethical standards outlined in the Declaration of Helsinki and the European Union’s General Data Protection Regulation (GDPR), ensuring both ethical conduct and stringent protection of personal data throughout the study and approval for the study was provided by the ethical committee of our institution.

### 2.2. Blood Pressure Measurement

Measurement to evaluate SBP was performed with the patient comfortably seated in a chair with arm support for 5 min before the measurement. The arm was supported and positioned at heart level. The patient was relaxed, with feet positioned flat on the floor, and had refrained from smoking, consuming caffeine, or engaging in physical exercise for at least 30 min prior to the measurement.

The cuff was selected based on the pre-measured circumference of the forearm. The cuff was positioned on the upper arm, approximately 2–3 cm above the antecubital fossa.

Systolic blood pressure value was determined by performing the average between two consecutive measurements at the right upper arm, each undertaken at a 2 min difference between them (rest period). If the difference between the first two consecutive measurements was larger than 10 mmHg, then a third measurement was performed after a rest period, and the blood pressure value was noted as the average of the last two measured values.

Blood pressure was measured in the same way in all visits using an Omron M6 Comfort device (OMRON, Kyoto, Japan) and recorded at each appointment.

### 2.3. Visit-to-Visit Systolic Blood Pressure Variability

Visit-to-visit SBP variability refers to the degree of fluctuation in SBP values over time, measured across multiple clinical visits. In our study, visit-to-visit SBP variability was evaluated over a period of three years at a three-month interval, with a maximum of twelve visits. 

SBP variability for each patient was defined in this study as the standard deviation (SD) of SBP recorded during the study. This metric captures the fluctuations in SBP values measured during consecutive visit-to-visit assessments. Calculating the SD quantifies the variability and provides an objective measure of the consistency or instability of SBP across time because it represents the average amount by which individual SBP readings deviate from the mean SBP value for that individual. 

### 2.4. Statistical Analysis

Descriptive statistics were used to summarize the demographic and clinical attributes of the study population. Continuous variables, including age and SBP variability, were expressed as mean and SD, while categorical variables, such as sex, smoking status, and comorbid conditions, were summarized as percentages. 

Logistic regression was performed to assess the association between SBP variability and stroke incidence with results reported as odds ratios (OR) with 95% confidence intervals (CI). Additional predictors, including diabetes, smoking, and chronic kidney disease, were taken in consideration in order to evaluate their collective contribution.

To explore time-to-event outcomes, Cox proportional hazards models were developed to assess the impact of SBP variability on stroke risk over the duration of the study. One model focused on overall stroke, while another explored different stroke subtypes, demonstrating the importance of SBP variability in relation to cerebrovascular outcomes. These findings underscored the clinical relevance of blood pressure patterns in predicting the likelihood of future events.

Analysis of survival and stroke-free probabilities was conducted using Kaplan–Meier plots, which classified individuals based on SBP variability ranges. 

All statistical procedures were conducted using RStudio Version 2024.12.0 and R Version 4.4.2. Statistical significance was considered for *p* < 0.05.

Approval for the study protocol was provided by the ethical committee of our institution.

## 3. Results

### 3.1. Study Population Description

The study included 208 patients. The study took place over a period of three years, with patient evaluations conducted quarterly. The average age of the study population was 65.32 years, demonstrating a somewhat uniform distribution group. The sex distribution was 46.64% females and 53.36% males. 

The population consisted of hypertensive men and women aged between 40 and 80 years old, with associated risk factors for cardiovascular disease (CVD). Dyslipidemia was present in 53.84% of patients, and 18.26% had diabetes mellitus. Additionally, 55.28% of patients were classified as obese, highlighting the prevalence of modifiable risk factors in this population. Smoking was observed in 40.38% of the patients. 

Regarding chronic health conditions, 13.94% of the patients had chronic kidney disease (CKD). Furthermore, 31.73% of the population had primary coronary disease (PCD), underscoring the cardiovascular risk profile of the group (Table 1).

### 3.2. SBP Variability Analysis

First, an analysis was performed to evaluate the distribution of various comorbid conditions and cardiovascular risk factors across the study population. 

This analysis shows the characteristics of patients grouped by SBP variability in three categories: low SBP variability, high SBP variability, and very high SBP variability. Patients in the high and very high-variability groups were generally older, with mean ages of 67.74 and 66.96 years, respectively, compared to 64.68 years in the low-variability group. Dyslipidemia prevalence increased markedly with SBP variability, affecting 50.31% in the low-variability group, 56.52% in the high-variability group, and 71.42% in the very high-variability group. Similarly, the prevalence of CKD and PCD was higher in the very high-variability group, with CKD present in 25% and PCD in 50% of these patients. Smoking prevalence was highest in the high-variability group (60.86%), suggesting a possible behavioral component contributing to variability (Table 2).

Logistic regression was performed prior to Cox proportional hazards analysis to provide an initial assessment of significant variables and their direction of effect. 

The logistic regression analysis in Table 3 revealed a statistically significant association between SBP variability and stroke incidence (*p* < 0.001). Based on the regression coefficient, each 1-unit increase in SBP variability was associated with a 65.6% increase in the odds of stroke. Systolic blood pressure variability demonstrates a significant association with the outcome, with an odds ratio (OR) of 1.72 (95% CI: 1.42–2.19, *p* < 0.001), indicating a 72% increase in odds per unit increase in variability. Average SBP with an OR of 1.03 (95% CI: 0.98–1.08, *p* = 0.172) reflects no significant effect. Other variables, including diabetes, sex, smoking, and chronic kidney disease, did not show significant associations (*p* > 0.05). Obesity demonstrated an OR = 3.04, *p* = 0.055, warranting further investigation.

Cox proportional hazards analysis was performed with different models in order to evaluate the association between SBP variability and different stroke risks. 

Model I was conducted to evaluate the association between SBP variability and stroke risk (ischemic and hemorrhagic) adjusted for different factors. SBP variability was found to be a significant predictor of stroke risk, with a hazard ratio (HR) of 1.55 (95% CI: 3.30–7.61; *p* < 0.001). This indicates that each 1-unit increase in SBP variability is associated with a 55% increase in stroke risk.

SBP average had an HR of 1.03 (95% CI: 2.67–2.95, *p* = 0.186), showing no statistical significance. Other variables, including age (HR: 0.97, *p* = 0.491) and sex (HR: 0.65, *p* = 0.452), showed no significant association. Similarly, smoking (HR: 0.82, *p* = 0.741), diabetes mellitus (HR: 0.25, *p* = 0.063), chronic kidney disease (CKD, HR: 1.52, *p* = 0.562), obesity (HR: 1.73, *p* = 0.303), dyslipidemia (HR: 0.66, *p* = 0.488) and primary coronary disease (HR: 0.51, *p* = 0.270) did not exhibit significant effects. (Table 4)

Model II revealed a significant association between SBP variability and the risk of hemorrhagic stroke and also identified a statistically significant association between SBP variability and the risk of ischemic stroke.

For hemorrhagic stroke, SBP variability was significantly associated with an increased risk, with an HR of 1.73 (95% CI: 0.19–2.80, *p* = 0.005), indicating a 73% higher risk per unit increase in variability. Similarly, for ischemic stroke, SBP variability was a significant predictor, with an HR of 1.21 (95% CI: 0.09–2.14, *p* = 0.031), corresponding to a 21% higher risk. When considering the overall risk of any stroke type, SBP variability was also significantly associated with an increased hazard, with an HR of 1.31 (95% CI: 0.08–3.38, *p* ≤ 0.001) (Table 5).

Our survivability analysis reveals that stroke-free and overall survival probabilities differ significantly across SBP variability groups, with the very high-variability group exhibiting the highest risk of stroke and mortality.

Stroke incidence increased markedly with higher SBP variability, as shown in Table 6. In the low-variability group, stroke events were limited to ischemic strokes (4.5%), with no hemorrhagic strokes observed. In contrast, the high-variability group had a higher proportion of both ischemic (17.4%) and hemorrhagic (8.7%) strokes. The very high-variability group demonstrated the highest overall stroke incidence (46.4%), including 28.6% ischemic and 17.9% hemorrhagic strokes. 

**Figure 1:**
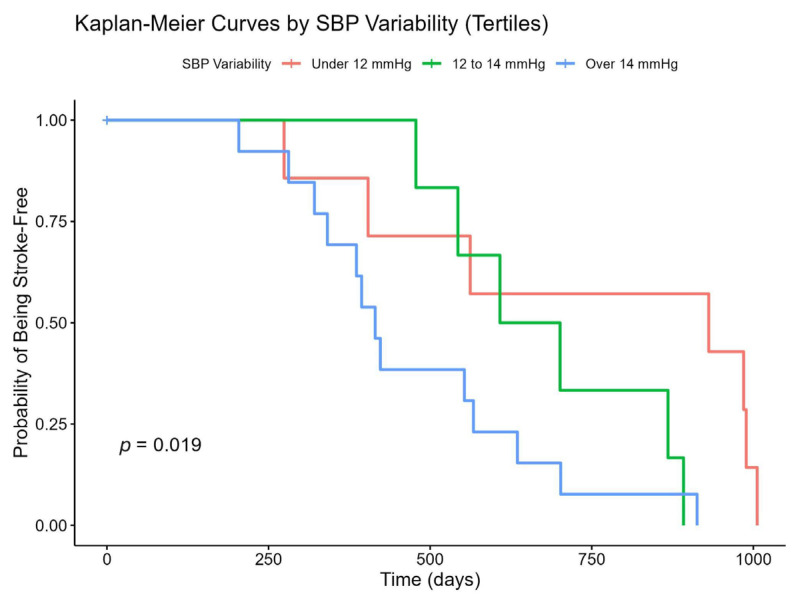
Impact of SBP variability on stroke-free survival. SBP: Systolic Blood Pressure; Red: low SBP Variability; Green: high SBP variability; Blue: very high SBP variability group.

Through our survivability analysis, the probability of remaining stroke-free over time is presented for three groups classified by SBP variability. The very high-variability group experiences stroke events earlier, suggesting an increased risk. By contrast, the high-variability group shows the most favorable stroke-free survival, while the low-variability group exhibits intermediate outcomes. The log-rank test *p*-value of 0.019 indicates a statistically significant difference among these survival curves (Figure 1).

**Figure 2:**
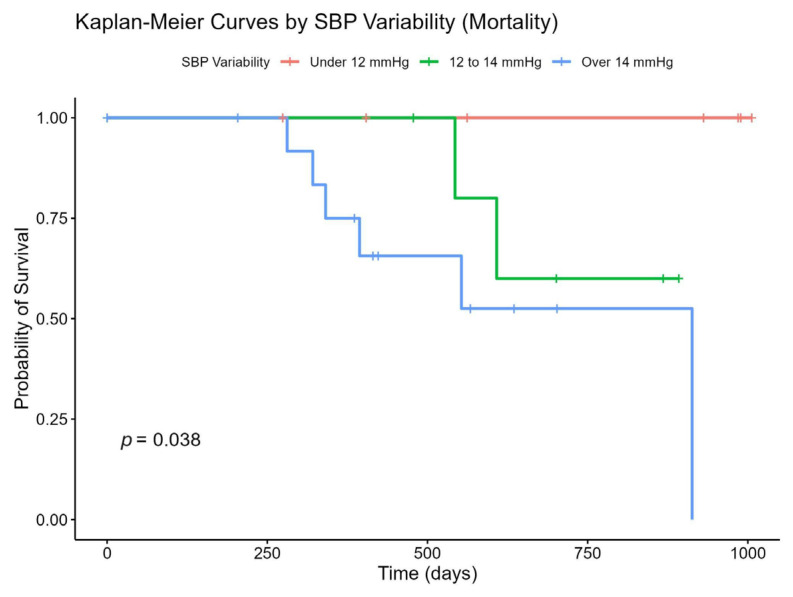
Association of SBP Variability and Mortality. SBP: Systolic Blood Pressure; Red: low SBP Variability; Green: high SBP variability; Blue: very high SBP variability group.

The probability of survival decreases most rapidly in the very high-variability group, demonstrating the highest mortality risk. Conversely, the low-variability group maintains a consistently high probability of survival, indicating lower mortality risk, while the high-variability group shows intermediate outcomes. The log-rank test *p*-value of 0.038 suggests a statistically significant difference in survival probability across the three groups (Figure 2). All recorded deaths in our study were of neurological causes, primarily due to fatal ischemic or hemorrhagic strokes and their complications.

## 4. Discussion

Our study shows that SBP variability serves as a robust predictor of stroke incidence, underscoring its important role in cerebrovascular risk. The findings revealed that higher SBP variability is strongly associated with an increased risk of both ischemic and hemorrhagic strokes, with the relationship being particularly pronounced for hemorrhagic stroke. Patients exhibiting greater fluctuations in SBP experienced significantly earlier stroke events and reduced stroke-free survival. Moreover, mortality rates were notably higher among individuals with very high SBP variability, indicating its profound impact on long-term outcomes. 

Our results indicate that patients exhibiting high and very high SBP variability were older on average (≥67 years). Results are consistent with Chowdhury et al. where it is shown that in an elderly hypertensive population (≥65 years), higher visit-to-visit SBP variability predicted stroke, myocardial infarction, heart failure, and composite cardiovascular events across ages 65–84 [17]. 

Dyslipidemia and CKD were more prevalent in the very high-variability group, supporting previous findings by Muntner et al. that highly unstable SBP variability can exacerbate endothelial dysfunction and accelerate vascular damage in individuals with lipid abnormalities or renal impairment [8].

In Lau et al., PCD was shown to have a significant increase in those with very high SBP variability, highlighting the interplay between fluctuating hemodynamics and ischemic cardiac pathology [18].

SBP variability emerged as a significant independent predictor of stroke risk, with both logistic regression and Cox proportional hazards analyses showing strong associations (72% increased risk). Each unit increase in SBP variability was linked to substantial increases in stroke odds and risk, whereas other variables, including age, sex, and comorbidities such as diabetes and chronic kidney disease, showed no significant effects. These results are consistent with evidence found by Rothwell et al. [13].

Additionally, Rothwell et al. have shown that visit-to-visit SBP variability is a powerful predictor of stroke independent of mean SBP, results consistent with our study [7]. Along with additional evidence from other recent reports, these findings support the importance of both lowering SBP and minimizing its fluctuations over time [19,20].

Our study revealed a significant association between SBP variability and the risk of hemorrhagic stroke and also identified a statistically significant association between SBP variability and the risk of ischemic stroke.

Through Cox proportional hazards analysis, we have shown that for hemorrhagic stroke, SBP variability was associated with a 73% higher risk per unit increase in variability. Similarly, for ischemic stroke, SBP variability was a significant predictor, with a 21% higher risk. When considering the overall risk of any stroke type, SBP variability was also significantly associated with an increased hazard, with a 31% increase in risk. These results are consistent with the ones found in recent medical literature. 

In a population-based cohort study from The Netherlands, Heshmatollah et al. found that higher SBP variability was associated with an increased risk of hemorrhagic and unspecified stroke [21]. In contrast, Rothwell et al. found that visit-to-visit SBP variability was more predictive of ischemic rather than hemorrhagic stroke in patients enrolled in the ASCOT-BPLA [7]. Finally, Shimbo et al. found that the association between SBP variability and stroke did not vary by stroke subtype, including both ischemic and hemorrhagic [12]. 

A study by Maulida et al. suggests that higher BP variability is linked to poorer clinical outcomes in lacunar and non-lacunar types of acute ischemic stroke, emphasizing the importance of monitoring BP variability in acute ischemic stroke management. Although in our study, we did not categorize stroke as lacunar or non-lacunar, this could be a future line of research regarding BP variability as a potential biomarker, taking into consideration the significant differences in the clinical presentations and pathophysiology of these subtypes, where lacunar strokes result primary from chronic hypertension and small vessel disease, while non-lacunar strokes are linked to thromboembolism or large-artery atherosclerosis [22].

The reason for these divergent results is currently unclear. Explanatory factors could include, among others, different populations examined, different study timeframes, and design study heterogeneity.

Our study showed a strong association between SBP variability and mortality risk. The very high-variability group exhibited the most pronounced decline in survival probabilities over time, underscoring the direct relationship between elevated SBP variability and increased mortality. The consistently lower mortality risk observed in the low-variability group suggests that stable blood pressure management may be a protective factor against fatal outcomes. Furthermore, the intermediate outcomes in the high-variability group reinforce the existence of a graded risk, wherein even moderate increases in SBP variability can adversely impact survival. Our results align with the findings from Muntner et al., which show that higher visit-to-visit SBP variability is associated with an increased risk of cardiovascular disease (CVD) and mortality, especially in the very high-variability group [23].

In the context of hypertensive patients, recent findings suggest that even modest increases in SBP variability can elevate the likelihood of cerebrovascular events, both ischemic and hemorrhagic [15,16].

We recognize several limitations in our study. First, the research primarily involved an elderly population with a mean age exceeding 65 years, restricting the applicability of the results to younger groups. Second, the relatively small sample size could limit the possibility of generalizing our findings, reduce statistical power, and constrain the ability to perform subgroup analyses. Third, the study did not account for the specific types of antihypertensive medications administered or examine the association between SBP variability and antihypertensive treatment. Finally, the study was conducted at a single center, which could limit the applicability of the findings to broader populations.

A possible line of inquiry for future research is the exploration of SBP variability as a prognostic biomarker in different stroke subtypes, particularly distinguishing between lacunar and non-lacunar ischemic strokes. Additionally, investigations into the interplay between SBP variability, arterial stiffness, and cerebral autoregulation may provide novel insights into modifiable targets for stroke prevention and management. 

## 5. Conclusions

Our study highlights the critical role of SBP variability as an independent predictor of stroke and mortality among hypertensive patients. 

Higher visit-to-visit SBP variability has shown an increased risk of stroke independent of other factors and was significantly associated with an increased risk of ischemic and hemorrhagic strokes.

Results demonstrated the graded relationship between SBP variability and mortality risk. The very high-variability group exhibited the most rapid decline in survival probabilities, while the low-variability group maintained a consistently high probability of survival. The intermediate-variability group showed an incremental risk, confirming the relationship between SBP variability and adverse outcomes. 

The findings underscore the importance of addressing SBP fluctuations alongside average SBP levels in clinical management, as stable blood pressure patterns may mitigate cerebrovascular and fatal cardiovascular outcomes.

## Figures and Tables

**Table 1:** Study population characteristics.

Characteristics	Values *n* = 208 (%)
Age *	65.32 (9.09) *
Sex	M: 53.36 F: 46.64
Smoker	40.38
Diabetes Mellitus	18.26
Primary Coronary Disease	31.73
Dyslipidemia	53.84
Obesity	55.28
Chronic Kidney Disease	13.94

* Age expressed in mean value and standard deviation in form Mean (SD).

**Table 2:** Patient characteristics by SBP variability.

Parameter	Low SBP Variability: SD of SBP < 12 (*n* = 157)	High SBP Variability: SD of SBP = 12–14 (*n* = 23)	Very High SBP Variability: SD of SBP > 14 (*n* = 28)
Age *	64.67 *	67.73 *	66.96 *
Dyslipidemia (%)	79 (50.31)	13 (56.52)	20 (71.42)
Obesity (%)	89 (56.68)	13 (56.52)	13 (46.42)
Smokers (%)	58 (36.94)	14 (60.86)	12 (42.85)
Diabetes Mellitus (%)	27 (17.19)	5 (21.73)	6 (21.42)
CKD (%)	17 (10.82)	5 (21.73)	7 (25)
PCD (%)	47 (29.93)	5 (21.73)	14 (50)

* Age expressed in mean value; SBP: Systolic Blood Pressure; SD: Standard Deviation; CKD: Chronic Kidney Disease; PCD: Primary Coronary Disease.

**Table 3:** Logistic regression analysis of risk factors and comorbidities.

Parameter	OR (95% CI)
SBP Variability	1.72 (1.42–2.19)
SBP Average	1.03 (0.98–1.08)
Age	1.06 (0.97–1.16)
Diabetes Mellitus	0.90 (0.20–3.45)
Sex (male)	0.58 (0.17–1.84)
Smoker	2.09 (0.66–6.93)
PCD	1.15 (0.35–3.57)
CKD	0.84 (0.17–3.53)
Obesity	3.04 (1.01–10.21)
Dyslipidemia	1.40 (0.45–4.59)

OR: Odds Ratio; CI: Confidence Interval; SBP: Systolic Blood Pressure; SD: Standard Deviation; CKD: Chronic Kidney Disease; PCD: Primary Coronary Disease.

**Table 4:** Association between stroke incidence and cardiovascular risk factors and other comorbidities.

Parameter	HR (95% CI)	*p* Value
SBP Variability	1.55 (3.30–7.61)	≤0.001
SBP Average	1.03 (2.67–2.95)	0.186
Age	0.97 (2.43–2.87)	0.491
Sex (male)	0.65 (1.24–7.15)	0.452
Smoking	0.82 (1.29–13.70)	0.741
Diabetes Mellitus	0.25 (1.06–2.94)	0.063
CKD	1.52 (1.44–567.85)	0.562
Obesity	1.73 (1.83–146.66)	0.303
Dyslipidemia	0.66 (1.23–8.10)	0.488
PCD	0.51 (1.16–5.40)	0.270

HR: Hazard Ratio; CI: Confidence Interval; SBP: Systolic Blood Pressure; CKD: Chronic Kidney Disease; PCD: Primary Coronary Disease.

**Table 5:** Association between SBP variability and risk of incidence of ischemic stroke, hemorrhagic stroke, and both types of strokes.

Outcome	HR (95% CI)	*p* Value
Hemorrhagic Stroke	1.73 (0.19–2.80)	0.005
Ischemic Stroke	1.21 (0.09–2.14)	0.031
Stroke (ischemic and hemorrhagic)	1.31 (0.08–3.38)	≤0.001

HR: Hazard Ratio; CI: Confidence Interval.

**Table 6:** Association of SBP variability, stroke type, and stroke incidence.

SBP Variability	Stroke Type	Stroke Incidence
Low (*n* = 157)	Ischemic (%)	7 (4.5)
	Hemorrhagic (%)	0 (0)
	Total (%)	7 (4.5)
High (*n* = 23)	Ischemic (%)	4 (17.4)
	Hemorrhagic (%)	2 (8.7)
	Total (%)	6 (26.1)
Very High (*n* = 28)	Ischemic (%)	8 (28.6)
	Hemorrhagic (%)	5 (17.9)
	Total (%)	13 (46.4)

SBP: Systolic Blood Pressure.

## Data Availability

Data are contained within the article.
